# Comparison of Synthetic vs. Biogenic Polymeric Process-Directing Agents for Intrafibrillar Mineralization of Collagen

**DOI:** 10.3390/polym14040775

**Published:** 2022-02-16

**Authors:** Neha Saxena, Joshua Mizels, Maegan A. Cremer, Vanessa Guarnizo, Douglas E. Rodriguez, Laurie B. Gower

**Affiliations:** 1 Department of Materials Science & Engineering, University of Florida, Gainesville, FL 32611, USA; nsaxena24@gmail.com (N.S.); jmizels1230@gmail.com (J.M.); mcremer@ufl.edu (M.A.C.); vguarnizo01@yahoo.com (V.G.); drodriguez@novabone.com (D.E.R.); 2 Bio-Therapeutics Drug Product Development, Janssen Pharmaceuticals, Inc., Malvern, PA 19355, USA; 3 Department of Orthopaedic Surgery, University of Utah, Salt Lake City, UT 84112, USA; 4 College of Medicine, University of Florida, Gainesville, FL 32611, USA; 5 Quality Engineering, Medtronic ENT, Jacksonville, FL 32611, USA; 6 R&D, Novabone Products LLC, Alachua, FL 32611, USA

**Keywords:** bone substitutes, biomimetic processing, mineralized collagen, intrafibrillar mineralization, osteopontin process-directing agent, polyaspartic acid

## Abstract

With the aging population, there is a growing need for mineralized tissue restoration and synthetic bone substitutes. Previous studies have shown that a polymer-induced liquid-precursor (PILP) process can successfully mineralize collagen substrates to achieve compositions found in native bone and dentin. This process also leads to intrafibrillar apatitic crystals with their [001] axes aligned roughly parallel to the long axis of the collagen fibril, emulating the nanostructural organization found in native bone and dentin. When demineralized bovine bone was remineralized via the PILP process using osteopontin (OPN), the samples were able to activate mouse marrow-derived osteoclasts to similar levels to those of native bone, suggesting a means for fabricating bioactive bone substitutes that could trigger remodeling through the native bone multicellular unit (BMU). In order to determine if OPN derived from bovine milk could be a cost-effective process-directing agent, the mineralization of type I collagen scaffolds using this protein was compared to the benchmark polypeptide of polyaspartic acid (sodium salt; pAsp). In this set of experiments, we found that OPN led to much faster and more uniform mineralization when compared with pAsp, making it a cheaper and commercially attractive alternative for mineralized tissue restorations.

## 1. Introduction

Researchers have been exploring ways to mineralize type I collagen scaffolds with hydroxyapatite, both to better understand biological mineralization processes and to create biologically resorbable bone substitutes. Previous work in our group has shown that the polymer-induced liquid-precursor (PILP) process can effectively mineralize collagen substrates up to and beyond levels of native bone (65 wt.% mineral) [1,2,3,4,5,6]. In the PILP process, first discovered for calcium carbonate [7,8,9], a polyanionic polymer is added to a supersaturated solution of mineral salts, leading to phase separation in the form of ion-sequestered nanodroplets that are stabilized in the hydrated amorphous form by the polymer. For the calcium phosphate system, these PILP nanodroplets can infiltrate collagen fibrils and later crystallize into hydroxyapatite with the [001] directions of the crystals roughly aligned with the long axes of the fibrils, as occurs in bone [1,2,4]. Although the mechanism of intrafibrillar mineralization remains controversial, Niu et al. [10] showed strong evidence that the Gibbs–Donnan effect (balancing of electroneutrality and osmotic equilibrium) provides the driving force for ion accumulation within the fibrils, while our group proposed that capillarity may also play a role in the transport of the liquid-like phase deep into the interstices of the fibrils [1]. Regardless of the mechanism, it is clear that by simply adding certain polyelectrolytes, the mineralization process is transformed in such a way as to generate an interpenetrating composite that is remarkably similar to the nanostructure of native mineralized tissues, such as bone [1,3,4,5] and dentin [11,12,13,14,15]. The key point for this paper is that simple anionic polypeptides (such as polyaspartic acid sodium salt, pAsp) can mimic the purported function of the highly charged, noncollagenous proteins (NCPs) found in mineralized tissues, which have long been thought to play a pivotal role in collagen mineralization in vivo [1,16].

One NCP that has been heavily studied is osteopontin (OPN), a member of the SIBLING (small integrin-binding ligand, N-linked glycoprotein) family of proteins that is abundant in bone and milk [17,18,19]. OPN is a ~34 kDa protein that is post-translationally modified, most notably containing many possible phosphorylation sites [18,20,21,22,23]. In addition, OPN contains a sequence of repeating aspartic acid residues [20,22]. These negatively charged groups are likely responsible for OPN’s affinity for binding Ca^2+^ [24,25,26] and/or mineral crystals [27]. Its high charge density also causes it (and many of the SIBLING proteins) to fall in the category of intrinsically disordered proteins (IDPs) [24,26,28], which is arguably why a simple polypeptide such as pAsp is reasonably effective at emulating this nonspecific ion-sequestering role of NCPs [1]. Although the function of OPN in mineralized tissues is debated, in vitro studies have shown that OPN inhibits hydroxyapatite formation in solution [29], but we have found that this inhibitory activity can actually promote intrafibrillar mineralization of collagen scaffolds [30]. It is also important to note that OPN is cleaved into smaller fragments in vivo [21,31,32]. For instance, in bone, OPN is a proteolytic substrate for PHEX (phosphate-regulating endopeptidase homolog X-linked), which is highly expressed by mineralizing cells and acts on the acidic serine- and aspartate-rich motif (ASARM) of SIBLING proteins [33]. In bovine milk, which is the source of the OPN used in our study, ~10% is in its full-length form (34 kDa), ~70% is 2/3 length (~23 kDa), and ~20% is fragments smaller than 23 kDa [21].

Previous work in our group has shown that bovine milk-derived OPN can be successfully used as a PILP process-directing agent for the mineralization of collagen scaffolds [30] under the same conditions first discovered with pAsp [1]. These experiments showed that mineralization of type I collagen sponges using OPN resulted in 75 wt.% mineral, with apatitic crystals that were highly aligned within the collagen fibrils [30]. An important aspect of intrafibrillar mineralization is that the intercalated crystals are extraordinarily small due to the confined space of the collagen fibrils within which they form, lending to the possibility that osteoclasts can resorb these metastable crystallites, as they do in bone. This was demonstrated in our prior study using EDTA-demineralized bone slices, which were then remineralized with OPN and pAsp (note that bone slices provide dense solid substrates, as are needed for osteoclast attachment via actin rings). We found that the OPN-remineralized slices were able to activate osteoclasts to a much greater extent than pAsp slices, with the number of actin rings comparable to those in the positive control of native bone [30]. We and others [34] have seen that the activation of osteoclasts occurs in an OPN-dependent manner. We believe that this enhanced activity is due to the presence of an RGD (cell-binding) motif present in OPN, which can interact with osteoclasts through the ανβ3-integrin (vitronectin receptor) [30,35,36,37]. This finding is highly significant because bone is a living tissue that is continually remodeled, and to do so, it must first be resorbed by osteoclasts before new bone can be laid down by osteoblasts, which, through intercellular communications, act in an orchestrated fashion via this bone multicellular unit (BMU) [23,38,39,40,41]. Therefore, OPN-directed mineralization of type I collagen substrates is a potential route for the creation of next-generation bone substitutes, where the bioactivity of OPN can potentially be used to trigger osteoclast resorption, which, when coupled with an appropriate osteoanabolic environment [42,43], should trigger concurrent osteoblast remodeling for the gradual replacement of the bone substitute, as occurs in native bone remodeling. This could provide significant advantages over the popular approach of biodegradable scaffolds, where resorption occurs through hydrolytic or enzymatic degradation. For that approach to be viable, the resorption rate needs to match the rate of bone ingrowth, both of which could differ among different people, bone locations, ages, disease states, etc. Our long-range goal is to make “biomimetic” bone, where the cells “see” a nanostructured material that “looks” like bone and a chemical composition that can be resorbed like bone, so that the cells can gradually remodel the synthetic bone via the BMU, as occurs during natural bone remodeling.

In this paper, we evaluate the differences in the mineralization of type I collagen substrates using pAsp or OPN in order to better elucidate the differences between synthetic versus biogenic biopolymer additives. In our previous studies, we noticed that the collagen substrates seemed to mineralize in a much shorter time frame when OPN was used [30,44]. Here, we quantified the mineralization kinetics, mineral location, precursor droplet size and concentration, and process-directing agent location in relation to the collagen substrate to determine if there are further advantages of using OPN for the processing of biomimetic bone substitutes.

## 2. Materials and Methods

### 2.1. Mineralization

Bovine-derived type I collagen tape (ACE Surgical Supply Company, Inc., Brockton, MA, USA) was cut into 1.5 × 1.5 cm squares prior to mineralization in 50 mM Tris buffer with 0.9% NaCl, 0.02% sodium azide, 4.5 mM CaCl_2_, 2.1 mM K_2_HPO_4_, and 50 µg/mL of either 27 kDa pAsp (n = 200, Alamanda^TM^ Polymers, Inc., Huntsville, AL, USA) or bovine-derived milk OPN mix (Lacprodan^®^ OPN-10, from Arla Foods Ingredients Group P/S, (Viby J, Aarhus, Denmark). Mineralization took place with three sponge pieces per 500 mL of mineralization solution at 37 °C with continual stirring for time points of 6 h, 12 h, 18 h, 1 d, 3 d, and 5 d, after which the specimens were rinsed in DI water for 30 min three times to remove buffer salts. Additional mineralization times of 2 h and 4 h were added for the OPN group. The specimens were then flash-frozen in liquid nitrogen and lyophilized for further characterization.

For kidney tissue mineralization, porcine kidneys were first decellularized according to the protocols described in Ross et al. [45]. The tissues were subsequently mineralized per standard PILP protocols (described above) using either 27 kDa pAsp or OPN mix (Arla Foods Ingredients Group P/S, Viby J, Aarhus, Denmark) [46]. The pAsp mineralization took place for 7–14 days, while OPN mineralization was run for 2–4 days. The different mineralization times were due to the observation of precipitation within the mineralization solution after a certain number of days, which is usually an indicator that full intrafibrillar mineralization has occurred, after which mineral will start to build up on the surfaces of the scaffolds. The specimens were then rinsed per the protocol described above and subsequently lyophilized.

### 2.2. X-ray Diffraction (XRD)

Samples were placed on a glass side for analysis with a X’Pert Pro powder X-ray diffractometer (PANalytical B.V. Almelo, Netherlands). Samples were scanned from 10° to 60° (2ϴ) with a step size of 0.01° and a dwell time of 10 s/step using Cu Kα X-rays (λ = 1.54 Å).

### 2.3. Thermogravimetric Analysis (TGA)

TGA was performed using a TGA/DSC 2 STAR system (Mettler-Toledo, Columbus, OH, USA) on sample amounts of 5–8 mg. A program was created to run the analyses from 25 to 600 °C with a heating rate of 20 °C/min and flow rates of 65 cc/min for Ar, 20 cc/min for O_2_, and 1 cc/min for CO_2_. Ash weights were determined by the mass remaining at 600 °C.

Mineral weight percentages (ash weights) from TGA were fit to the Avrami equation:

(1) $$\frac{Y}{Y_{\max}}=1-\exp\left(-{\mathrm{Kt}}^{n}\right)$$

using MATLAB. In this equation, Y is the mineral amount in weight percentage, Y_max_ is the maximum mineral amount possible, t is time, and K and n are Avrami constants [47]. R^2^ values for the linearized fits for this model were 0.91 and 0.98 for OPN and pAsp specimens, respectively.

### 2.4. Scanning Electron Microscopy (SEM) and Energy Dispersive X-ray Spectroscopy (EDS)

Freeze-dried samples were mounted on aluminum stubs with double-sided copper tape. All samples were sputter-coated with amorphous carbon before analysis with either an FEI Nova 430 (4 h OPN sample) or an FEI XL-40 (Thermo Fisher Scientific, Hillsboro, OR, USA) (remaining samples), operated at 15 kV and with a spot size of 3.

### 2.5. Fibril Diameter Analysis

Fibril diameters were measured using Image J’s line and measurement tools on SEM micrographs taken at 20,000· magnification. Three micrographs were analyzed per group (both specimen interiors and exteriors), and 20 measurements were taken per micrograph.

### 2.6. Transmission Electron Microscopy (TEM) and Electron Diffraction

Early time point mineralization samples (4 h OPN and 18 h pAsp) were fixed overnight at 4 °C with 4% paraformaldehyde in PBS. Samples were then dehydrated through a series of graded ethanol solutions and embedded in acrylic resin (LR White Hard, Electron Microscopy Sciences, Hatfield, PA, USA) for sectioning. Ultrathin sections (~90 nm) were cut using a Leica Ultracut T (Leica Biosystems, Buffalo Grove, IL, USA) at a rate of 8 mm/s using a diamond knife. Sections were collected on carbon-coated 400 mesh copper TEM grids (Electron Microscopy Sciences, Hatfield, PA, USA) and imaged with a Hitachi 7600 TEM (Hitachi High-Tech Analytical Science America, Inc., Westford, MA, USA). at an accelerating voltage of 80 kV.

Five-day mineralization samples were pulverized in liquid nitrogen and dispersed in ethanol. Ethanol solutions were dropped onto 400-mesh copper grids (Electron Microscopy Sciences, Hatfield, PA, USA) coated with amorphous carbon films, with the grids placed atop filter paper. The samples were analyzed with a JEOL 200CX TEM (JEOL, Peabody, MA, USA) at an accelerating voltage of 200 kV.

Kidney tissues mineralized with either OPN or pAsp were fixed in paraformaldehyde as described above and embedded in LR White Hard resin. Ultrathin sections (~70 nm) were cut as described above, stained with uranyl acetate (2%), and analyzed with a Hitachi 7600 TEM (Hitachi High-Tech Analytical Science America, Inc., Westford, MA, USA) at 80 kV.

### 2.7. Fluorescent Tagging and Imaging

A 2 × 2 cm^2^ piece of collagen tape (ACE Surgical) was hydrated with deionized water and then pulverized in liquid nitrogen. After pulverization, the collagen powder was resuspended in DI water, and 100 μL of this collagen suspension was placed on a glass-bottom Petri dish (Chemglass Life Sciences, Vineland, NJ, USA) and air-dried overnight.

For conjugation of process-directing agents to fluorophore, a fluorescein-5-isothiocyanate (FITC)-based fluorophore (Alexa Fluor 647) was purchased from ThermoFisher Scientific (Waltham, MA, USA). FITC was solubilized in N,N-dimethylformamide (DMF) (Sigma-Aldrich, St. Louis, MO, USA) at 5 mg/mL. Both the Lacroprodan^®^ OPN-10 (Arla Foods, Viby J, Aarhus, Denmark) and 27 kDa pAsp (Alamanda Polymers, Huntsville, AL, USA) were solubilized in bicarbonate buffer at 5 mg/mL. For OPN conjugation, 100 μL of FITC solution was slowly added to 1000 μL of OPN/bicarbonate solution while vortexing. For pAsp conjugation, 500 μL of FITC solution was slowly added to 500 μL of OPN/bicarbonate solution while vortexing. The conjugations took place at room temperature for two hours on a rocker plate. To purify, Micro Bio-Spin™ P-6 Gel Columns and Tris Buffer were purchased from Bio-Rad. For conjugated pAsp, 40 μL of FITC/pAsp (plus 20 μL bicarbonate buffer) was added to each spin column. For conjugated OPN, 22 μL of FITC/OPN (plus 38 μL bicarbonate buffer) was added to each spin column. The spin columns were then centrifuged at 1000 g for four minutes.

To confirm conjugation, purified samples of labeled process-directing agents were examined using UV/vis spectroscopy (ThermoFisher Scientific, Waltham, MA, USA). The ratio of each sample’s absorbance at 495 nm (FITC excitation) to number of moles (A495/mol) was calculated for each process-directing agent: A495/mol OPN = 8.69 × 10^7^ and A495/mol pAsp = 4.24 × 10^7^.

After successful conjugation, 2 mL of each process-directing agent was used for a 60-min PILP mineralization of pulverized, adsorbed collagen in glass-bottom Petri dishes. Control groups were conducted using 2 mL of each process-directing agent in Tris buffer to observe the interaction between OPN or pAsp and collagen in the absence of calcium and phosphate ions. After the 60-min experiment, collagen was labeled with Invitrogen AlexaFluor^®^ 647 NHS ester (succinimidyl ester) (ThermoFisher Scientific, Waltham, MA, USA), a bright far-red fluorescent dye. Briefly, 100 μg of AlexaFluor^®^ was solubilized in 10 μL DMF. The AlexaFluor/DMF solution was then added to 90 μL of bicarbonate buffer. An equal amount of this solution was added to each Petri dish, and then 500 μL of bicarbonate was added to each Petri dish to cover the entire glass surface with conjugation solution. After one hour of conjugation at room temperature, each Petri dish was thoroughly rinsed with deionized water. The specimens were then air-dried overnight. After drying, a drop of ProLong^®^ Gold Antifade Mountant (Molecular Probes, ThermoFisher Scientific, Eugene, OR, USA) was added to each Petri dish and allowed to cure overnight at room temperature.

Spinning-disk confocal microscopy was performed on an Olympus DSU-IX81 (Olympus Corporation, Shinjuku City, Tokyo, Japan) to analyze the specimens. The FITC-labeled process-directing agents were analyzed using a FITC filter set, and the AlexaFluor^®^ 647-labeled collagen samples were analyzed using a CY5 filter set. All images were taken using a 60· water immersion objective. Negative control samples (without fluorophores) were imaged using the same exposure time and gain to confirm the absence of autofluorescence of the specimens in both channels.

### 2.8. Nanoparticle Tracking Analysis

Conventional (no polymer), 27 kDa pAsp, and OPN mineralization solutions (see protocol above) were prepared and immediately analyzed with a NanoSight LM10 (Malvern PANalytical, Salisbury, United Kingdom) at 37 °C for 60 s (n = 3) using a 405 nm wavelength laser. The nanoparticle tracking data were analyzed by deleting the “FALSE” (incorrectly tracked) particles and using only correctly tracked particles to determine particle sizes. Particle concentrations were taken from the NanoSight software and statistical significance between groups was determined via one-way ANOVA and Tukey’s post hoc tests with a cutoff *p*-value of 0.05.

## 3. Results

### 3.1. XRD and TGA Analysis of Mineralization Kinetics

**Figure 1:**
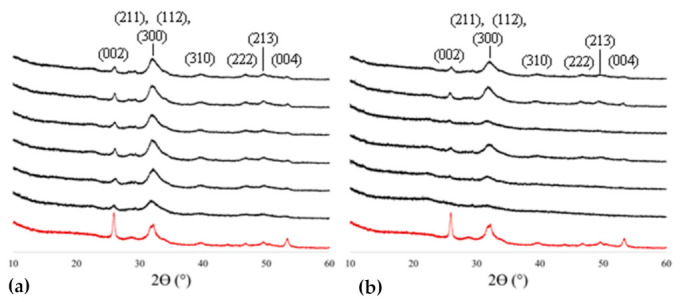
XRD patterns of collagen sponges mineralized with OPN (**a**) or pAsp (**b**), and with bovine bone for comparison (red curve). As expected, the patterns match hydroxyapatite (indices noted above peaks), and the peaks are broad from the nanocrystalline size that results from constrained intrafibrillar mineral (as seen in bone). But in comparison, there are relatively sharper peaks at all time points for the OPN-mineralized sponges, while peaks for the pAsp-mineralized sponges don’t fully emerge until after ~18 h of mineralization. From bottom to top, time points are: 6 h, 12 h, 18 h, 1 d, 3 d, and 5 d.

X-ray diffraction (XRD) was used to compare the mineralization progression of the OPN mix (Figure 1a) versus 27 kDa pAsp (Figure 1b) at 6 h, 12 h, 18 h, 1 d, 3 d, and 5 d time points and showed that the emergence of hydroxyapatite peaks of the mineralized sponges occurred faster with OPN (Figure 1). All specimens exhibit XRD patterns with broad peaks, indicative of nanocrystalline/impure apatite, as seen in native bone (shown by the red curve). Sponges mineralized using pAsp exhibited little mineral content at early time points. Peak widths generally decrease with increasing mineralization time in pAsp samples but are always a little broader than those in OPN samples.

**Figure 2:**
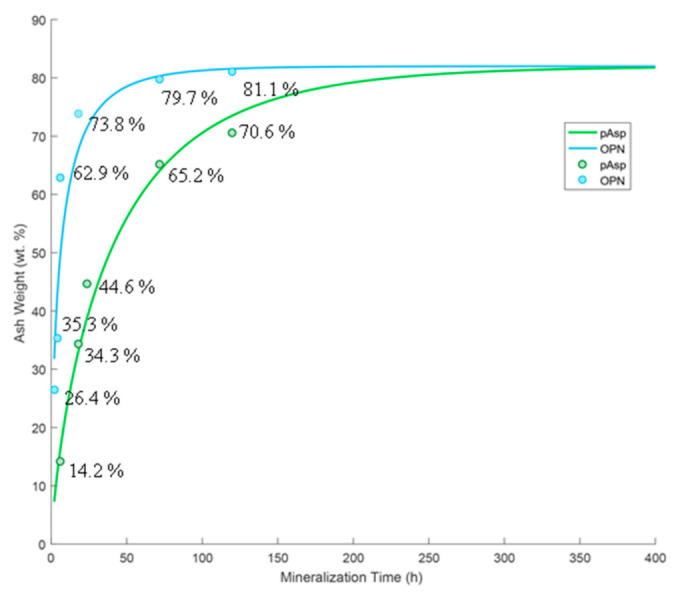
TGA ash weights at 600 °C of 2 h, 4 h, 6 h, 18 h, 3 d, and 5 d time points of OPN-mineralized sponges (blue), and 6 h, 18 h, 1 d, 3 d, and 5 d time points of pAsp-mineralized sponges (green), fit to Avrami kinetics curves. All ash weight values have a standard instrument error of ±0.1 wt.% and a standard error in the mineralization process of ±2.0 wt.%.

Figure 2 shows thermogravimetric analysis (TGA) ash weights at 600 °C with data fit to Avrami kinetic curves. A large amount of mineral was present in OPN-mineralized sponges at early time points (62.9 ± 2.1 wt.% ash at 6 h), while pAsp specimens exhibited a slower rate of mineralization, with only 14.2 ± 2.1 wt.% ash at 6 h and 65.2 ± 2.1 wt.% at 3 days. Note that all of these values are artificially higher by about 13.5% due to the argon gas used for TGA, which does not fully combust the organics (see Discussion section).

### 3.2. Microscopy Analysis of Mineralized Fibril Morphologies and Textures

**Figure 3:**
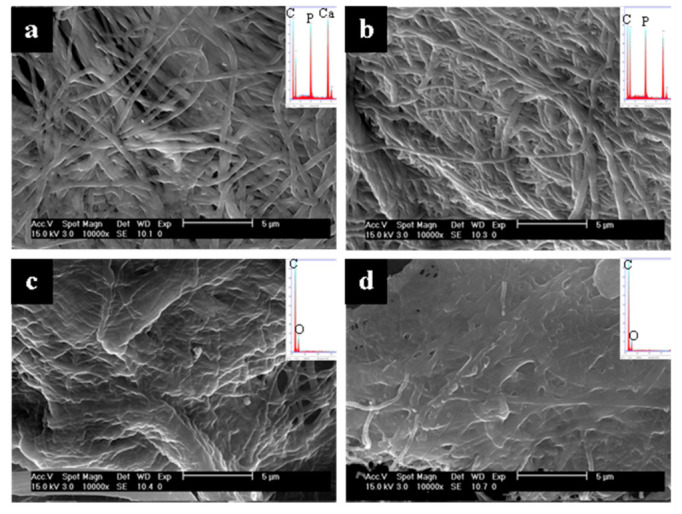
SEM micrographs of the 6 h time point of collagen sponges mineralized with OPN (top) or pAsp (bottom), showing the surfaces (**a**,**c**) and interiors (**b**,**d**) of the sponges. A high degree of mineralization was achieved using OPN on both the surface and interior (insets show large Ca/P peaks relative to C and O peaks), but little to no mineral is seen for either when using pAsp. Note the difference in texture between the two, where OPN samples have ‘plumped up’ fibrils from the intrafibrillar mineral, while the pAsp samples have fibrils that have collapsed into a mat upon drying.

Representative SEM micrographs and EDS spectra of the surfaces and interiors of collagen sponges mineralized with either OPN or pAsp at different time points are shown in Figure 3 (and other time points in the Supplementary Materials, Figures S1–S3). Figure 3 shows a dramatic difference at the 6 h time point in the comparison of the OPN- versus pAsp-mineralized sponges. Judging by the EDS spectra, the fibrils in both the interior and exterior of the sponge mineralized using OPN appear uniformly infiltrated with mineral to a relatively high degree (Figure 3a,b insets). Although EDS is only semiquantitative with such rough specimens, one can still compare the sizes of the Ca and P peaks (mineral) relative to the C peak (organic), which shows roughly equivalent sizes, as is typical of native bone. When pAsp was used, little to no mineral is apparent in either the exterior or interior of the specimen at this early time point (Figure 3c,d). The smooth and flat texture of the pAsp fibrils is also clearly different from the “plumped up” OPN fibrils that became embedded with mineral and did not collapse upon drying. OPN even showed the presence of mineral at the 4 h time point, with substantial intrafibrillar mineral in the outer region of the sponge (Figure S1a) but much less mineral in the interior of the cross-sectioned sponge (Figure S1b). Although this is an early mineralization time point (corresponding to 35.3 ± 2.1 wt. % ash in Figure 2), the fibrils appear relatively uniform in diameter (Figure S1a).

**Figure 4:**
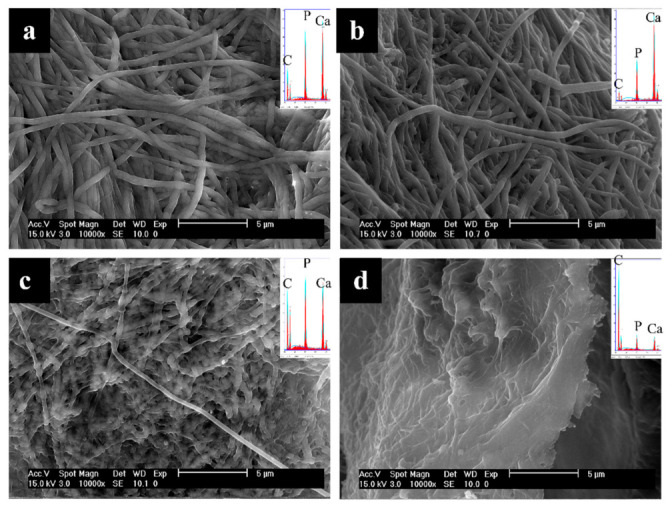
SEM micrographs of the 18 h time point of collagen sponges mineralized with OPN (top) or pAsp (bottom), showing surfaces (**a**,**c**) and interiors (**b**,**d**) of the sponges. A high degree of mineralization was achieved throughout the sponge using OPN (insets show very large Ca/P peaks relative to C and O peaks), but mineralization was mostly confined to the surface when pAsp was used. A highly nodular texture can be seen for the fibrils in (**c**) at this intermediate stage of mineralization.

After 18 h of mineralization using OPN (Figure 4), fibrils appear larger in diameter than at the 6 h time point, and overall mineral content has increased, as surmised from EDS peak heights, which now have Ca/P peaks that are significantly larger than the C peak (Figure 4a,b insets). ImageJ analysis of fibril diameters measured in SEM micrographs shows that collagen fibrils in the 6 h OPN group were 310 ± 81 nm, and those in the 18 h OPN group were 417 ± 101 nm (statistically significantly different from one another with *p* < 0.0001). On the exterior of the sponge mineralized using pAsp at this 18 h time point, intrafibrillar mineral is present mostly in the form of lumps or nodular regions in the fibrils, and EDS spectra indicate relatively high mineral content in these areas (Figure 4c inset). When compared with the 6 h time point of OPN mineralization (Figure 3a,b inset), EDS shows that the sample at the 18 h time point with pAsp has similar mineral content (Figure 4c inset) but has a nodular morphology instead of more uniformly expanded fibrils. The interior of the sponge mineralized for 18 h using pAsp shows only a small degree of mineralization, as seen by fibril flatness and EDS analysis (Figure 4d).

When mineralized for 3 days (Figure S2), the exterior of the OPN-mineralized sponge still exhibits morphologies indicative of high amounts of intrafibrillar mineral, and the EDS spectrum indicates a very high degree of mineralization overall. In the interior, however, less mineral is present, and a slightly nodular morphology is apparent in this area (Figure S2b), although this was rarely observed in the OPN groups. After mineralization with pAsp for 3 days (Figure S2c,d), the exteriors of the sponges exhibit morphologies indicative of both intra- and interfibrillar mineral (fibrils appear to be full of mineral at the nodular regions and cemented to each other via a mineral coating, respectively) (Figure S2c). The interior of the sponge is less highly mineralized, and the mineral appears to still be mostly intrafibrillar, with a nodular morphology present in some areas, mainly where fibril thickening is more pronounced (Figure S2d). For OPN, the 5-day mineralization time (Figure S3) shows very little difference as compared to the 3-day sample; large amounts of intrafibrillar mineral are observed on both the exteriors and interiors of the sponges at both time points (Figures S2 and S3a,b, respectively). This agrees with TGA data (79.7 ± 2.1 vs. 81.1 ± 2.1 wt.% for 3 and 5 days, respectively). For pAsp, a modest increase in mineral contents is seen between the 3- and 5-day mineralization times via TGA (65.2 ± 2.1 vs. 70.6 ± 2.1 wt.%), but the morphologies are similar except that the nodules appear to have smoothed out a bit at the later time point (Figures S2 and S3c,d, respectively). In general, there appears to be stronger mineral confinement (less interfibrillar cement) within the fibrils when OPN was used as a process-directing agent.

**Figure 5:**
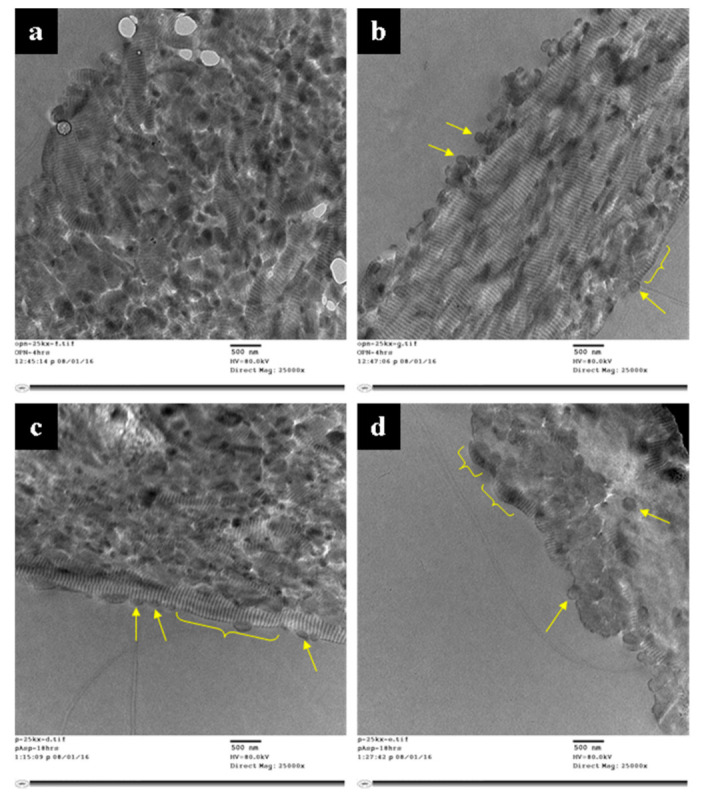
TEM micrographs of unstained, embedded and microtomed 4 h OPN samples (**a**,**b**), and 18 h pAsp samples (**c**,**d**). Both samples show the start of mineral deposition in the gap zones of the fibrils, as seen by the marked banding patterns. Fibril cross-sections (arrows) indicate primarily intrafibrillar mineral, but the lack of streaks in longitudinal sections suggests the mineral is still amorphous. OPN samples show relatively uniform fibril diameters, while some bulges are evident in pAsp samples (yellow brackets).

Figure 5 shows representative TEM micrographs of early mineralization time points (~35 wt.% ash at 600 °C) of sponges mineralized with OPN for 4 h (top) and pAsp for 18 h (bottom). Both groups show preferential mineral deposition in gap zones within the fibrils at these early time points, as evidenced by the pronounced banding pattern, which is apparent even though these samples were not stained (other than by infiltration of electron-dense mineral precursor). The biggest difference between the two groups is the greater apparent presence of nodular morphologies in the pAsp group, which was also seen in SEM. The bulges also appear darker, supporting the premise that these regions contain more mineral content.

**Figure 6:**
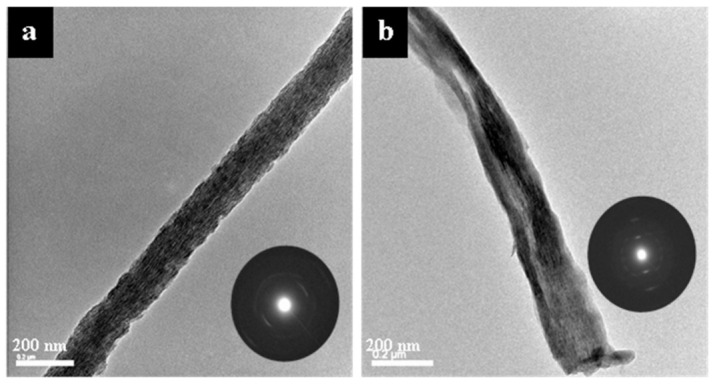
TEM micrographs and electron diffraction patterns of the 5 d time point of collagen sponges mineralized with OPN (**a**) and pAsp (**b**). Both OPN and pAsp resulted in highly aligned, intrafibrillar apatite crystals. The crystals (dark streaks) in image (**b**) in the mid region of the fibril appear quite long as compared to those in image (**a**), and the SAED pattern suggests a high degree of alignment, yet the crystals are clearly not incorporated uniformly throughout the fibril.

Figure 6 shows representative TEM micrographs and electron diffraction patterns of the late-stage, 5-day time point of collagen mineralized with either OPN (Figure 6a) or pAsp (Figure 6b). Both OPN and pAsp result in intrafibrillar, [001]-aligned apatitic mineral. Mineralization with OPN also appears to result in more uniform mineral distribution with what appears to be shorter intrafibrillar crystals than when pAsp was used (Figure 6a vs. Figure 6b).

### 3.3. NanoSight Nanoparticle Tracking Analysis

**Figure 7:**
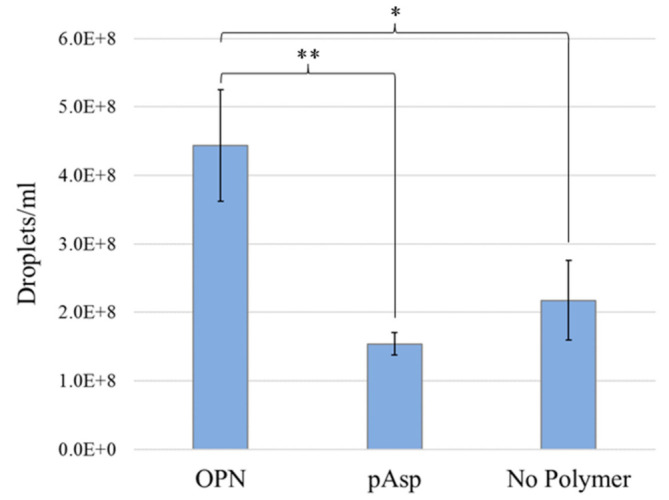
Droplet concentrations (×10^8^) based on NanoSight analysis showing that the droplet concentration in the OPN group was statistically significantly larger than the two other groups. No statistically significant difference is seen between the concentration of droplets in the pAsp group and the group without process-directing agent. * *p* < 0.05, ** *p* < 0.01.

To determine if the faster mineralization kinetics observed in the OPN group as compared with the pAsp group was due to more or larger precursor droplet sizes in the OPN mineralization solution, nanoparticle tracking analysis was conducted on mineralization solutions containing OPN, pAsp, or no process-directing agent. Droplet sizes measured between the groups are not statistically different from one another (Table 1), although they do visually appear quite different in the videos showing scattering from the droplets moving in solution (Supplementary Materials, Videos S1–S3). The droplets appear to drastically change in shape as they move in solution, indicating that they are most likely liquid-like in character. In addition, because these droplets are above 40 nm in diameter, if they were solid particles, one would see a bluish tint to the solution due to Rayleigh scattering. However, these solutions remained clear for multiple days when they contained a process-directing agent versus a few hours if they did not, suggesting that the droplets are liquid and not solid. Although droplet sizes do not significantly differ between groups, droplet concentrations are significantly different (Table 1 and Figure 7). Figure 7 shows that droplet concentrations in the OPN solutions are much higher (2–3×) than those in pAsp or conventional solutions.

### 3.4. Fluorescence Analysis of Polymer–Collagen Interactions

We hypothesized that the faster mineralization kinetics in the OPN group might be due to a greater interaction between OPN and collagen since it reportedly has a collagen-binding domain [23,25,48,49,50,51], so we examined the interactions between the two process-directing agents and collagen using fluorescent labeling. Supplemental Figures S5 and S6 show representative images from these experiments. Figure S5 shows projected confocal fluorescence images of z-stacks of pulverized collagen incubated with Tris buffer (no Ca^2+^ or PO_4_^3−^) and either OPN (top) or pAsp (bottom) for 1 h but without mineral salts. Fine fibrillar detail can be observed in all four images, indicating that both polymers can bind to the collagen fibrils without having to be incorporated with Ca^2+^/PO_4_^3−^ mineral precursors. In the images showing the FITC-labeled process-directing agent, there are dark borders around the collagen pieces that appear to be depletion zones, particularly for pAsp, which appears to have more polymer bound to the glass substrate than OPN (Figure S5d vs. Figure S5b, respectively). Figure S6 shows projected confocal fluorescence images of z-stacks of pulverized collagen mineralized with either OPN (top) or pAsp (bottom) PILP solutions for 1 h. The images with the AlexaFluor^®^ label show fine fibrillar detail (Figure S6a,c). The FITC channel (b and d), however, shows much blurrier images than the AlexaFluor^®^ (a and c) or the FITC channel when just Tris buffer was used in the control (Figure S5b,d). The image showing FITC-labeled OPN shows some fluorescence corresponding to areas with collagen, but there are also concentrated bright spots on some areas of the collagen (Figure S6b). The image showing FITC-labeled pAsp in the presence of PILP solution (Figure S6d), however, is very similar to the image showing FITC-labeled pAsp when just Tris was used (Figure S5d), except the overall image appears blurrier. Here, there appears to be much more of the process-directing agent on the glass substrate when pAsp was used as compared with OPN (Figure S6d vs. Figure S6b, respectively). Some individual (non-stacked) fluorescence images can be found in Supplemental Figures S7 and S8.

## 4. Discussion

The presence of nanocrystalline apatitic mineral was confirmed in all measured specimens (Figure 1), albeit much less at the early time points for pAsp-mineralized collagen. The differences in relative peak intensities in comparing XRD patterns between bovine bone and mineralized sponge can be attributed to differences in texture due to the alignment of collagen fibrils within lamellae in bone versus a lack of higher-order organization of collagen fibrils within the sponges; in this particular case, the enhanced (002) peak in the bone sample shows that the slice happened to be cut with a preferential orientation along the [001] of hydroxyapatite [2,47].

Figure 2 shows ash weight percentages at 600 °C at various time points of OPN- and pAsp-mineralized sponges. It is important to note that while ash weights have traditionally been used to estimate mineral weight percentages in mineralized collagen composites [1,52], the numbers here are not directly comparable because of the gas flow used in the TGA instrument. When TGA of unmineralized collagen was performed under these same conditions (with argon flow), an ash weight of 13.5 ± 2.1% was obtained at 600 °C, indicating that full combustion of organics is dependent on the gas (our past studies were done in air, which seems to fully combust the organics [1,52]). This indicates that the specimen mineralized with pAsp for 6 h (ash weight of 14.2 ± 2.1% at 600 °C) likely contains little to no mineral, which is consistent with SEM micrographs and EDS spectra (Figure 3c,d). The TGA data points were fit to Avrami kinetic curves using a maximum weight percentage of mineral of 82.0 wt.% for both groups. Although these curves appear to fit well, the Avrami constants are n = 0.5788 and K = 0.3279 for the OPN group and n = 0.7806 and K = 0.0541 for the pAsp group. In an ideal situation, the n constants should be between 1 and 6 [47], which was not the case here. This is because these measurements do not track a material’s transformation (i.e. crystal nucleation and growth); rather, our interest was in comparing the uptake kinetics of mineral precursors into the collagen scaffold. This relies on both the transport of precursor droplets to the fibrils and then infiltration into fibrils, both of which could be influenced by the polymer process-directing agent, as discussed below. One should keep in mind that the transport factor is also markedly influenced by other factors that must be kept constant in these types of kinetics studies, such as solution volume (which contains a given amount of precursor droplets dictated by the supersaturation/polymer stabilizer) relative to the matrix volume that takes up the precursor, as well as stirring or other methods that bring the droplets to the collagen matrix more rapidly, such as flow perfusion [53].

From SEM micrographs and EDS spectra (Figure 3 and Figure 4 and Figures S1–S3), it is clear that mineralization occurs more quickly using OPN than pAsp. In addition to this difference in kinetics, OPN-mineralized specimens generally have more uniform fibril diameters at earlier time points than those found in pAsp-mineralized specimens, which exhibit a more nodular morphology (Figure 4c, Figures S2 and S3c,d). This nodular texture has been noticed in our previous pAsp studies [6,52], and while it does seem to smooth out as the fibrils become fully infiltrated with mineral, it was interesting to see that the process-directing agent can have a pronounced effect on this phenomenon. Another important difference is that all of the OPN-mineralized specimens appear to contain mostly intrafibrillar mineral, even though very high degrees of mineralization had occurred by the 5-day time point; in contrast, the pAsp-mineralized specimens showed some interfibrillar cement by day 3 (Figure S2c). This trend toward higher degrees of intrafibrillar mineral using OPN has also been observed using other substrates, such as decellularized porcine kidney tissue (Supplementary Figure S4) [46]. In these TEM micrographs showing interstitial (collagen-based ECM) tissue, the OPN-mineralized tissue shows distinctly demarcated mineralized fibrils that constrain the crystallites (Figure S4a,b), while the pAsp-mineralized tissue shows crystals that splay outward and do not appear to be constrained by the fibrils (Figure S4c,d). Interestingly, a diffuse electron-dense globular substance was present on some of the fibrils mineralized with pAsp, which may suggest that the excessive overgrowth of crystals occurred from large accumulations of the PILP phase (Figure S4d).

TEM micrographs of early mineralization time points (~35 wt.% ash) reveal similar nanostructures between the two groups (Figure 5). Dark bands corresponding to gap zones within the collagen fibrils are considered indicative of intrafibrillar mineral, as these specimens are unstained. As described in the literature, gap zones are often observed to fill with mineral first [54] in both bone [55] and in vitro model systems [56,57,58]. In addition, some circular structures are present (arrows), which are likely cross-sections of fibrils since they are ~150–200 nm in diameter. The biggest difference between the two samples is the presence of more and larger bulges (brackets) in the pAsp sample than in the OPN sample, which is consistent with the nodular structures observed in the SEM micrographs (Figure 4c).

At later stages, TEM micrographs and electron diffraction patterns of unstained, pulverized 5-day specimens show crystal nanotextures similar to those found in native bone, with striations from [001]-oriented nanocrystals whose axes are roughly aligned parallel to the long axes of the associated collagen fibril (Figure 6). We proposed that this crystallographic alignment might arise from the confinement of growing crystals within the interstices of the fibrils such that the rapid growth direction becomes dominant [1], the concept of which has been nicely illustrated using track-etch filtration membranes that provided confinement of hydroxyapatite growth [59] and recent tomographic imaging of pAsp-mineralized collagen itself [58]. The diffraction pattern arcs are typical of mineralized collagen (and bone) due to the angular spread of crystallites that presumably follow the tilt axis of the twisted microfibrillar bundles that constitute collagen fibrils [60]. Interestingly, while the early-stage mineral incorporation appeared similar for both additives, both showing dark diffuse (amorphous) bands (Figure 5), the later stages showed a markedly different texture as the mineral became more fully incorporated throughout both the gap and overlap zones of the fibrils (Figure 6). It appears as though the crystals may be more preferentially deposited within gap zones in the OPN-mineralized fibrils than in the pAsp-mineralized fibrils, resulting in a more uniform appearance of intercalated mineral. This seems consistent with the observations of Tay and colleagues, who showed that phosphate-containing molecules promote preferential mineralization of the gap zones in early stages, which they referred to as hierarchical mineralization [56,61,62], as seen in bone. The more uniform crystallite size may also suggest that OPN can serve to promote nucleation of the crystallites via a templating effect, as suggested by Tay and colleagues for their small phosphate-containing molecules [62,63]. This may not necessarily be from an epitaxial type of relationship (popularly hypothesized in the older literature) but could just result from the high-charge character of the phosphate groups of the molecules, which may become bound to the collagen in specific regions. For example, molecular dynamics simulations suggest that charged side chains located in gap zones may alter the density of bound water in these zones, thereby lowering the enthalpic penalty for ion desolvation and thus nucleation [64].

As indicated in Table 1, droplet sizes obtained from nanotracking analysis were about the same for all three groups. However, it is important to note that a 405 nm laser was used in these experiments, and droplets/particles less than ~20 nm in diameter are unable to be detected with this system. Our prior dynamic light scattering (DLS) studies found that the PILP droplets start off smaller than this (14–19 nm) [6], and OPN is known to stabilize even smaller nanoclusters [65]. In the NanoSight scattering videos, the particles do visually appear smaller for the OPN solution (see Supplementary Videos S1 vs. S2 and S3 ). These data were obtained upon immediate mixing of solutions and therefore may not be representative of equilibrium conditions, which may take time to evolve as the polymer gradually sequesters ions (or stable ion clusters [65,66] or a liquid-condensed phase (LCP) [67]). Nevertheless, the droplets were clearly present and could be visualized with this technique, and their concentrations could be measured. The term “droplet” is used, even for the solution containing no polymer, because there is evidence that there may be an LCP in these mineral salt solutions as well, even without polymer additive [67]. In addition, in Supplemental Videos S1–S3, the droplets move in similar fashions, appearing to change shape in all three solutions, and the solutions remain visually clear for a few hours. However, the droplets of LCP in solutions without a process-directing agent differ somewhat and are apparently not stable long enough to diffuse to collagen fibrils and infiltrate them in any substantial amount, which is why the solution becomes turbid within hours as homogeneous crystal nucleation and growth occur [6,52]. In contrast, when a process-directing agent (such as OPN or pAsp) is added, the droplets are stabilized for longer [66], which we call the PILP phase, allowing them time to diffuse to the collagen fibrils, where they can infiltrate the fibrils. The droplet stability can also be inferred by the solution stability, which is stable for 2–4 days for OPN solutions and 4–7 days for pAsp solutions, after which the solutions become visually cloudy due to conventional precipitates. These precipitates may form on the surfaces of collagen scaffolds in the form of large extrafibrillar crystallites [1], as seen in much of the literature prior to this discovery, but apparently cannot infiltrate into the intrafibrillar compartments, which is thought to be because they are either too large or too solidified.

Based on NanoSight data, there are ~2.88· more droplets in the early stages of OPN solution than in the early stages of pAsp solution (Figure 7). As suggested by Azuma et al. the OPN mix used in these experiments likely consists of ~10% 34 kDa macromolecules, ~70% 23 kDa macromolecules, and ~20% smaller fragments [21]. The commercial pAsp is 27 kDa and considered monodisperse. Therefore, it is assumed that there is approximately the same number of macromolecules of the process-directing agent in both solutions. The reason for the difference in droplet number may arise from something else, such as the likely existence of both negative and positive fragments in the OPN mix due to OPN’s negative and positive domains, and these may be able to stabilize different types of droplets [10,20]. The polydispersity of the molecular weights may also allow the OPN mix to somewhat stabilize precursor droplets better than the relatively monodisperse pAsp because there would be more molecules to interact at surfaces of droplets. Another possible reason that the OPN mix may stabilize more droplets than pAsp is that OPN may exhibit some secondary structure [68] (due to its negative, positive, and hydrophobic domains). Computational predictions suggest that although OPN is a highly disordered structure, it may form some short α-helices, and/or it may change its conformation upon binding to collagen [23]. In addition, OPN contains many phosphorylation sites, some of which are immediately adjacent to each other. The pKa of phosphate groups is lower than that of carboxylic acid side chains (2.2 versus 3.86), which could cause greater interaction with Ca^2+^ ions, particularly if calcium bridges form between adjacent phosphate groups. It is perhaps the greater number of droplets in the early stages of OPN solution than in the early stages of pAsp solution that contributes to the increase in mineralization kinetics observed in these experiments. This difference in droplet concentration may change over time (only short times were measured), particularly if droplets grow by coalescence, which is known to occur for less stable PILP systems, such as CaCO_3_ [69]. 

However, if one examines the diffusion flux of droplets in both solutions based on data collected via nanotracking analysis, it is clear that these differences in droplet number and size do not fully explain the faster mineralization kinetics observed using OPN as compared with pAsp (a full analysis is provided in the Supplementary Material section on “Diffusion Flux Analysis”). Thus, we considered other differences between these two polymer systems. For example, the OPN mix contains some smaller fragments, and they may be able to infiltrate collagen fibrils if they are less than 5.7 kDa [70]. According to the Gibbs–Donnan theory suggested by Niu et al. [10], however, this would actually reduce the driving force for mineral infiltration. However, given the variably charged domains on OPN, the fragments could interact with Ca^2+^, PO_4_^3−^, and OH^−^ ions, bringing the ions in close proximity to one another to promote faster hydroxyapatite formation. If crystallization of the amorphous phase occurs more quickly, this would then reduce the internal ion concentration and thus promote drawing more ions into the fibrils, and so on. 

It is also possible that OPN droplets have a higher affinity for collagen fibrils than pAsp droplets do, or perhaps OPN droplets are more likely to be drawn into the fibrils. It is likely that the OPN droplets do interact more with collagen due to OPN’s specific collagen-binding domain [25,48,49,50,51]. In addition, the mixture of positively and negatively charged domains on OPN fragments would allow them to interact with both positively and negatively charged groups on collagen. As mentioned earlier, this increase in affinity for collagen could also contribute to the uniformity of mineralization along collagen fibrils, leading to more uniform diameters (less nodular) in the early stages of mineralization when using OPN, which is not observed when pAsp is used (Figure 4c and Figure S1). To test this theory, experiments were conducted in which collagen was mineralized in the presence of fluorescently labeled OPN or pAsp.

Unfortunately, the results of the fluorescence experiments were not able to answer the initial question about why there are differences in the uniformity of the collagen fibril diameter at the early stages of mineralization between the two groups due to the low resolution of optical microscopy. However, these studies did show some interesting trends. In both groups, the images of FITC-labeled process-directing agents showed much clearer labeling along individual fibrillar bundles when calcium and phosphate were not present in the solution than when they were added to form PILP solutions (compare Figure S5 vs. Figure S6). This may indicate that when calcium and phosphate are not present, the process-directing agents are able to interact with the collagen itself due to charge interactions between the OPN or pAsp and amine-containing side chains on collagen molecules. When calcium and phosphate are present, this interaction likely still occurs, but process-directing molecules may undergo complexation with each other via the mineral precursor phase, which makes the images appear more diffuse and blurry in the FITC channel (Figure S6b,d). Another interesting finding was that, in all cases, there was more process-directing agent on the glass substrates when pAsp was used than when OPN was used (Figures S5 and S6). Even though both glass and pAsp have a negative surface charge, apparently, there is some surplus of pAsp that does not attach to the collagen and thus lands on the glass, unlike OPN, which is known to contain a specific collagen-binding domain [23,48,51]. To test whether these results were not just a factor of the pAsp being more attracted to the glass substrate than OPN, the fluorescence experiment was repeated without the presence of collagen. After one hour of incubation in either Tris or PILP solution, the FITC-labeled OPN was much more intense on the glass substrate than the FITC-labeled pAsp (Supplementary Figure S9). This is likely due to the fact that the surface charge of glass is negative [71] and OPN contains positive domains [20], while pAsp does not (aside from the N-terminus, which is presumably attached to FITC in these experiments). This confirms that OPN is not less attracted to the glass substrate in the initial experiments but instead is more attracted to the collagen than pAsp is. 

Another difference between OPN and pAsp is the presence of bright spots in the FITC channel when OPN was used. In the initial 1 h time point experiment, this trend was only seen in the specimens exposed to PILP solution (Figure S6b). To determine the source of these bright spots, an additional experiment was conducted in which a larger solution volume of Tris buffer was used (20 mL instead of 2 mL) and a longer time point was examined (6 h instead of 1 h) to examine both polymers without mineral ions. One can see that these bright spots also occurred in the OPN samples incubated in only Tris in this experiment (Supplementary Figure S10). Therefore, these bright spots do not indicate preferential association of the process-directing agent with mineral but rather seem to indicate complexation of the process-directing agent with itself (flocculation). It makes sense that this was seen more in the OPN samples than in the pAsp ones because the OPN mix likely contains fragments with different charges that could easily associate with one another if they have opposite charges, as opposed to the uniformly negatively charged pAsp.

Overall, it seems that the variation in kinetics and uniformity of mineral precursor infiltration may be a colloid issue. In fact, Gower recently suggested that a better terminology for the nonclassical crystallization mechanisms observed in a variety of biomineralizing systems might be Colloid Assembly and Transformation (CAT) [72]. For example, in the system here, one has to consider how the polymer influences the size and stability of the colloids (PILP droplets), as well as how the colloids interact with the matrix, which then influences their amorphous-to-crystalline transformation. Certainly, the polymer type and molecular weight are known to influence the colloid stability [6,65], and as suggested by the globular accumulations in the pAsp-mineralized kidney tissue [46], it seems that pAsp PILP droplets are less stable and more prone to coalescence growth than OPN PILP droplets (Figure S4d). Thus, we believe that more and smaller droplets are stabilized by OPN, and with OPN’s specific binding interactions with collagen, the accretion of precursor droplets along the length of the fibrils is more spread out, thereby resulting in both the enhanced kinetics and uniformity of mineral infiltration.

## 5. Conclusions

In this study, we observed differences in PILP mineralization processes of type I collagen using OPN and pAsp as process-directing agents. Of particular interest is the faster mineralization kinetics seen when OPN was used as compared to pAsp. In addition to the synthesis of bone substitutes, we consider that OPN can also effectively remineralize dentin lesions, so faster kinetics could make clinical translation easier in the dental clinical setting. Another consideration is the cost. Given the fact that this bovine milk-derived OPN mix is available in bulk quantities for a much cheaper price than pAsp and is in clinical trials for use as a supplement in infant formula [73], the use of an OPN mix to create commercial products could be a more commercially viable way to create mineralized tissue for bone substitutes. Using OPN, the collagen fibrils were more uniformly mineralized, even at low levels of mineralization (~35 wt.% ash), and the crystals appeared to be more intrafibrillar and perhaps shorter in length. Based on these studies, we infer that OPN’s ability to create more precursor droplets at early time points, which are also more stabilized, as well as its greater affinity for both collagen and different ion types, contributes to these differences, particularly the faster mineralization kinetics and perhaps the larger amounts of intrafibrillar mineral. The uniformity of mineral infiltration, which was far superior with OPN, could also be very important if one wants to vary the degree of mineralization in order to tailor the mechanical properties. For example, one could not simply use a shorter mineralization time or a lower mineral salt reactant concentration to lower the mineral content and thus the modulus if nodular regions were disrupting the interconnectivity of the desired interpenetrating composite. Further studies are needed in this area to determine how these differing mineral textures influence the mechanical properties of mineralized collagen scaffolds. Ideally, this should be carried out with dense and organized collagen scaffolds [4,5] if one hopes to achieve mechanical properties similar to bone.

As we indicated in our prior paper [30], it is clear that OPN is multifunctional, influencing both the mineralization process as well as the bioactivity of the final product. The present study suggests that it is even more complicated than this because these process-directing agents can also influence both the degree of intra- versus interfibrillar mineral, the nanoscale texture and uniformity of the mineral within the fibrils, the size of the crystallites, and the interfibrillar glue, each of which will likely have a pronounced impact on the mechanical properties. Clearly, further studies are needed to better understand these vitally important process-directing agents, and there are many other NCPs that could be examined as well. This in vitro model system has proved invaluable for helping elucidate these complexities of the biomineralization of collagenous tissues. We hope that it will ultimately help us control all of these factors that simultaneously influence both the structure and therefore the properties, as well as bioactivity, of these bone-like constructs. From a tissue engineering perspective, we believe that this biomimetic processing approach for preparing biomimetic bone is the most promising avenue toward achieving next-generation bone substitutes that are able to retain their load-bearing capacity throughout the BMU remodeling process.

## Figures and Tables

**Table 1:** ACP precursor droplet sizes and numbers obtained from NanoTracking Analysis. No statistical difference in droplet sizes was observed between groups. Solutions containing the OPN mix had more droplets than solutions with pAsp or solutions without a process-directing agent.

Sample	Droplet Size (nm)	Droplet Concentration (per mL)
OPN Mix	81 ± 41	4.44 × 10^8^ ± 0.82 × 10^8^
27 kDa pAsp	92 ± 50	1.54 × 10^8^ ± 0.16 × 10^8^
No Polymer	83 ± 49	2.17 × 10^8^ ± 0.58 × 10^8^

## Data Availability

Not applicable.

## Supplementary Materials

The following supporting information can be downloaded at: https://www.mdpi.com/article/10.3390/polym14040775/s1, Figure S1: SEM micrographs of the 4 h time point of collagen sponges mineralized with OPN, showing the surface of the sponge (a) and interior of the sponge that was cut in half (b). Figure S2: SEM micrographs of the 3-d time point of collagen sponges mineralized with OPN (top) or pAsp (bottom), showing surfaces (a & c) and interiors (b & d) of the sponges, Figure S3: SEM micrographs of the 5-d time point of collagen sponges mineralized with OPN (top) or pAsp (bottom), showing surfaces (a & c) and interiors (b & d) of the sponges, Figure S4: TEM micrographs of embedded, microtomed, and stained (2% UA) decellularized porcine kidney interstitial tissue that has been PILP-mineralized with OPN (top) and pAsp (bottom), Figure S5: Projected views of confocal fluorescent z-stack images of collagen tagged with AlexaFluor^®^ 647 (left) and process-directing agent tagged with FITC (right), after incubation in Tris buffer solution for 1 h, Figure S6: Projected views of confocal fluorescent z-stack images of collagen tagged with AlexaFluor^®^ 647 (left) and process-directing agent tagged with FITC (right), after incubation in PILP solution for 1 h, Figure S7: Individual confocal fluorescent images of collagen tagged with AlexaFluor^®^ 647 (left) and process-directing agent tagged with FITC (right) after incubation in PILP solution for 1 h, Figure S8: Individual confocal fluorescent images of collagen tagged with AlexaFluor^®^ 647 (left) and process-directing agent tagged with FITC (right) after incubation in PILP solution for 1 h, Figure S9: Epifluorescence images of glass slides incubated with FITC-tagged process-directing agent incubated in either Tris buffer (left) or PILP solution (right), Figure S10: Projected views of confocal fluorescent z-stack images of collagen tagged with AlexaFluor^®^ 647 (left) and process-directing agent tagged with FITC (right) after incubation in a larger volume (20 mL) of Tris buffer solution for 6 h. Video S1: OPN droplets visualized with NanoSight particle tracking analysis, Video S2: pAsp droplets visualized with NanoSight particle tracking analysis, Video S3: Droplets without polymer stabilizer visualized with NanoSight particle tracking analysis.
