# Supplementary material for: Comparison of Synthetic vs. Biogenic Polymeric Process-Directing Agents for Intrafibrillar Mineralization of Collagen
Source: Polymers (Basel). 2022 Feb 16;14(4):775. doi: 10.3390/polym14040775 (PMC8879695; doi:10.3390/polym14040775)
Supplement: Supplementary file 1 [file polymers-14-00775-s001.zip › polymers-1588747-supplementary.pdf]

Comparison of synthetic vs. biogenic polymeric process-directing agents for intrafibrillar mineralization of collagen

Neha Saxena [1,2], Joshua Mizels [1,3], Maegan A. Cremer [1,4], Vanessa Guarnizo [1,5], Douglas E. Rodriguez [1,6], and Laurie B. Gower [1*]

Supplementary Material

This Supplementary Material contains a section providing a quantitative "Diffusion Flux Analysis" of the NanoSight particle size/concentration data, to help elucidate the variations of kinetics of collagen mineralization observed for OPN versus pAsp. It also provides SEM/EDS micrographs of the other timepoints in the series of mineralization times; 4 hours (Figure S1), 3 days (Figure S2) and 5 days (Figure S3). It also contains TEM data from a prior paper on the mineralization of decellularized kidney tissue (Figure S4), which shows supportive evidence that helps explain the reason why OPN yields more intrafibrillar mineral than pAsp. Figures S5 – S9 are data relevant to the fluorescence microscopy study of polymer-collagen interactions. Lastly, Videos S1 – S3 provide visualization of the NanoSight nanoparticle tracking analysis data and illustrate the nanodroplet sizes and changes in shape.

Diffusion Flux Analysis:

To better assess the kinetic variation between pAsp vs. OPN, the diffusion flux was estimated from the particle size and concentration data provided by the NanoTracking Analysis. For simplicity, we model the droplets as particles diffusing across a distance x, representing half the thickness of the specimen (1 mm), where we know the initial concentration of droplets is zero. We assume any effects of pores within the specimen that would be small enough to affect

the droplet diffusion are negligible since the droplets are much smaller than the average pore size within the scaffolds. From Fick's law:

$$J = -D\frac{dc}{dx} = -D\frac{(c_2 - c_1)}{(x_2 - x_1)} = -D\frac{(0 - c)}{(0.001 - 0)} = D\left(\frac{c}{0.001}\right)$$

where J is diffusion flux, D is diffusion coefficient, and c is droplet concentration. For the diffusion of particles in a dilute solution, D can be estimated by the Stokes-Einstein equation.{Cussler, 1997 #269}:

$$D = \frac{kT}{6\pi\mu r}$$

Where k is the Boltzmann constant, T is temperature, μ is solvent viscosity, and r is particle radius. We can assume k, T, and μ to be the same for both solution types, giving the difference in diffusion coefficients to be completely dependent upon the droplet radii. This simplifies our diffusion flux equations to (in SI units):

$$J_{OPN} = \frac{D}{40.5 \times 10^{-9}}\left(\frac{4.44 \times 10^{14}}{0.001}\right) = 1.10 \times 10^{25} D \ \frac{droplets}{m^2 \cdot s}$$

and

$$J_{pAsp} = \frac{D}{46 \times 10^{-9}}\left(\frac{1.54 \times 10^{14}}{0.001}\right) = 3.35 \times 10^{24} D \ \frac{droplets}{m^2 \cdot s}$$

Therefore,

$$J_{OPN} = 3.28 J_{pAsp}$$

However, this does not complete the picture because, in addition to droplet diffusion to the fibrils, an increase in collagen mineralization also relates to the volume of each droplet as droplets will eventually solidify and then crystallize into hydroxyapatite. So, we must also compute the average droplet volume. Assuming a spherical shape:

$$V = \frac{4}{3}\pi r^3$$

Therefore,

$$V_{OPN} = \frac{4}{3}\pi(40.5 \times 10^{-9})^3 = 2.78 \times 10^{-22}\ m^3$$

and

$$V_{pAsp} = \frac{4}{3}\pi(46 \times 10^{-9})^3 = 4.08 \times 10^{-22}\ m^3$$

Therefore,

$$V_{pAsp} = 1.47V_{OPN}$$

So, barring any other effects, the mineralization kinetics of the OPN solution can be given by:

$$K_{OPN} = \frac{3.28}{1.47}K_{pAsp} = 2.23K_{pAsp}$$

However, the OPN solution resulted in optimal mineralization content (~65 wt.% ash at 600 °C) after just 6 hours of mineralization, while the pAsp solution took 12x longer to achieve this ash weight (72 hours of mineralization). In addition, if we consider early time points, such as after 6 hours of mineralization, the OPN solution produced samples with 63 wt.% ash whereas the pAsp solution resulted in samples with only 14 wt.% ash.

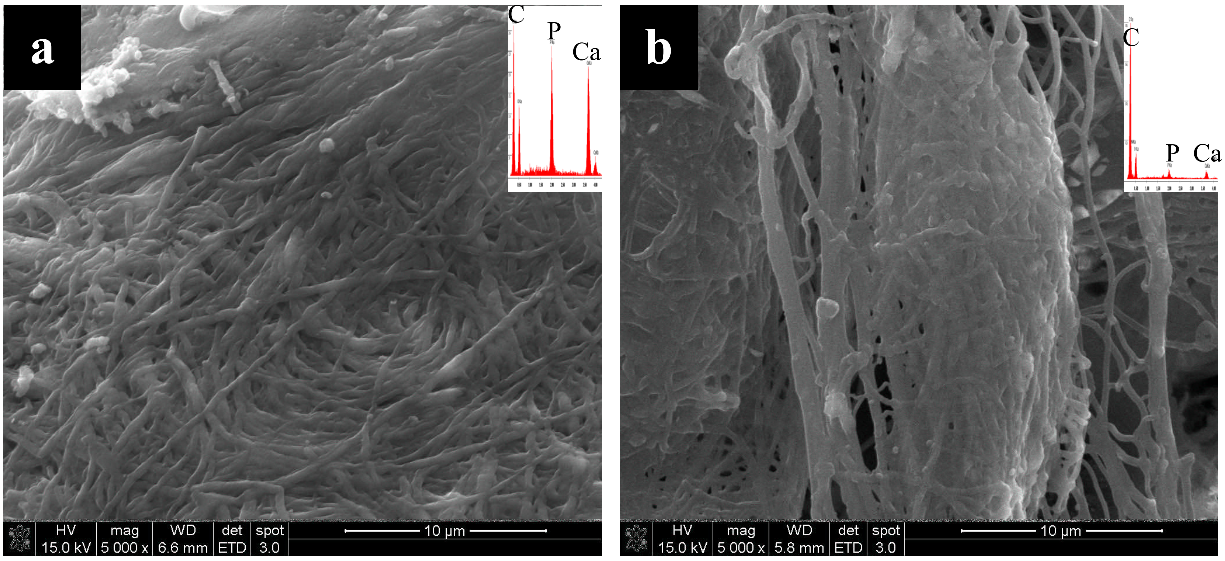

Figure S1. SEM micrographs of the 4 h time point of collagen sponges mineralized with OPN, showing the surface of the sponge (a) and interior of the sponge that was cut in half (b). Both images show relatively uniform (non-nodular) fibril diameters. Mineralization appeared to be intrafibrillar, but very little had reached the interior of the sponge at this time point, as seen by the very small Ca and P speaks in EDS (inset).

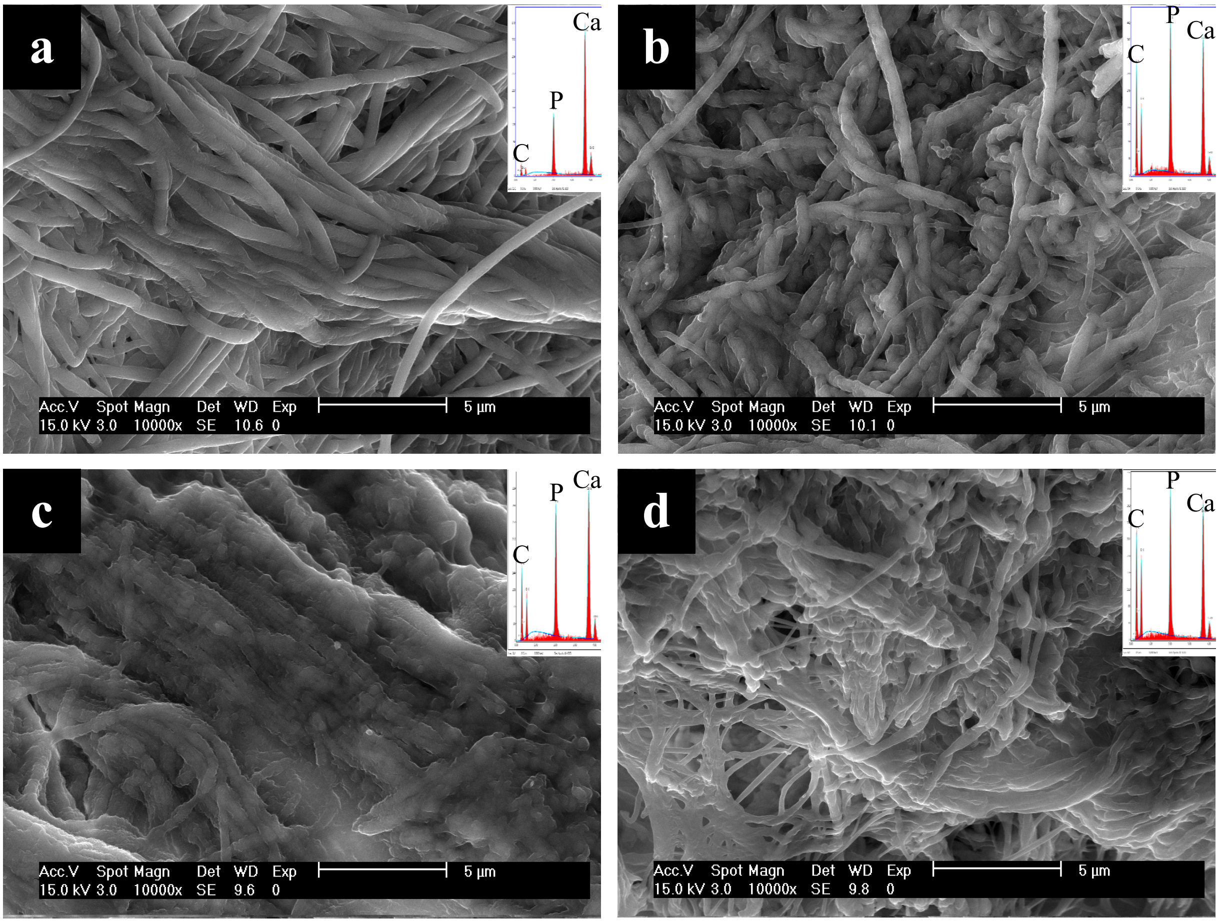

Figure S2. SEM micrographs of the 3-d time point of collagen sponges mineralized with OPN (top) or pAsp (bottom), showing surfaces (a & c) and interiors (b & d) of the sponges. Sponges mineralized with OPN exhibited primarily intrafibrillar mineral, while those mineralized with pAsp exhibited some extrafibrillar mineral on the surfaces of the sponges. The interior of the pAsp sample showed more uniformity of mineral along the fibrils (less nodular).

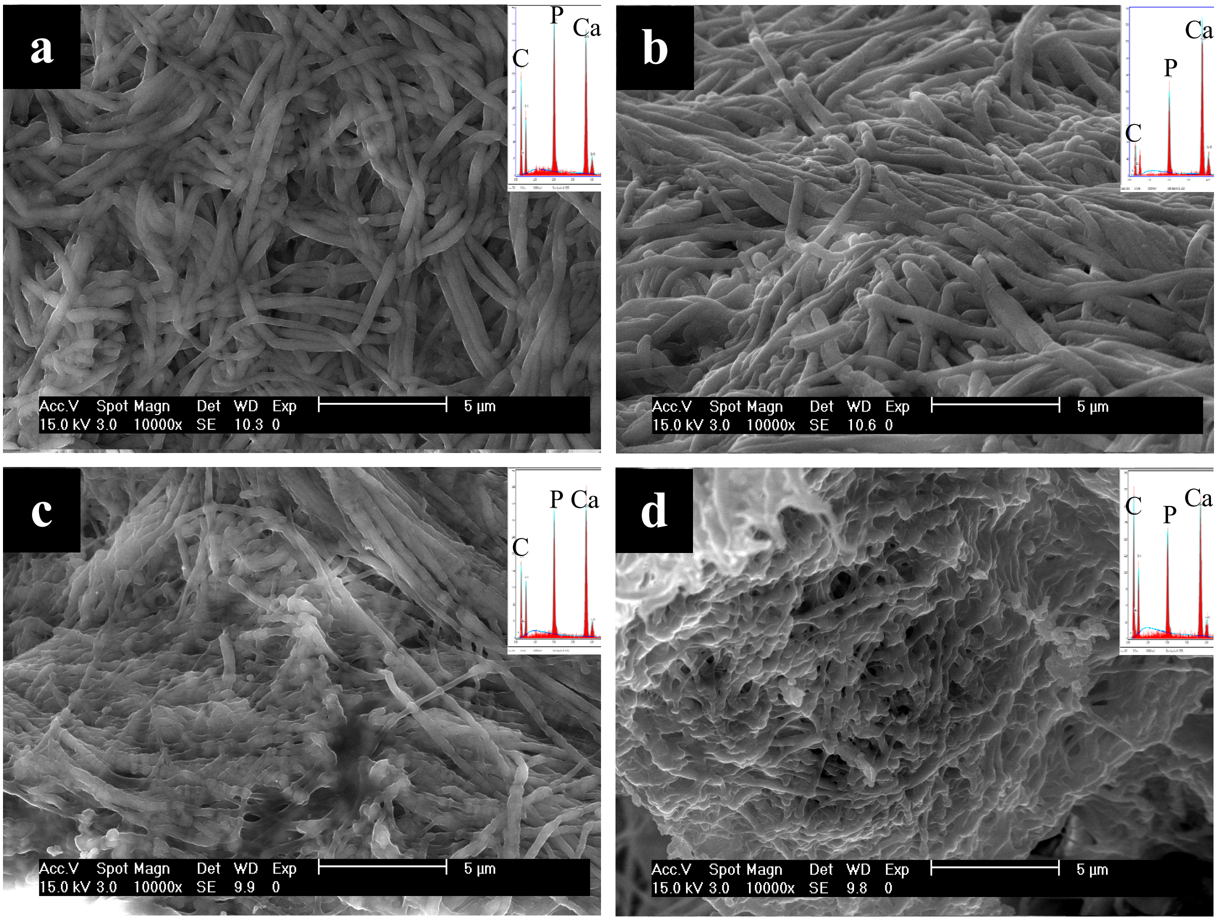

Figure S3. SEM micrographs of the 5-d time point of collagen sponges mineralized with OPN (top) or pAsp (bottom), showing surfaces (a & c) and interiors (b & d) of the sponges. Sponges mineralized with OPN exhibited primarily intrafibrillar mineral, while those mineralized with pAsp exhibited some extrafibrillar mineral on the surfaces of the sponges, which appears as an interfibrillar coating (i.e., not spherulitic) in (c). Note the distinct difference in fibril diameters, which have become considerably larger for the OPN-mineralized sponge (SEM magnifications are equivalent throughout the whole set of figures).

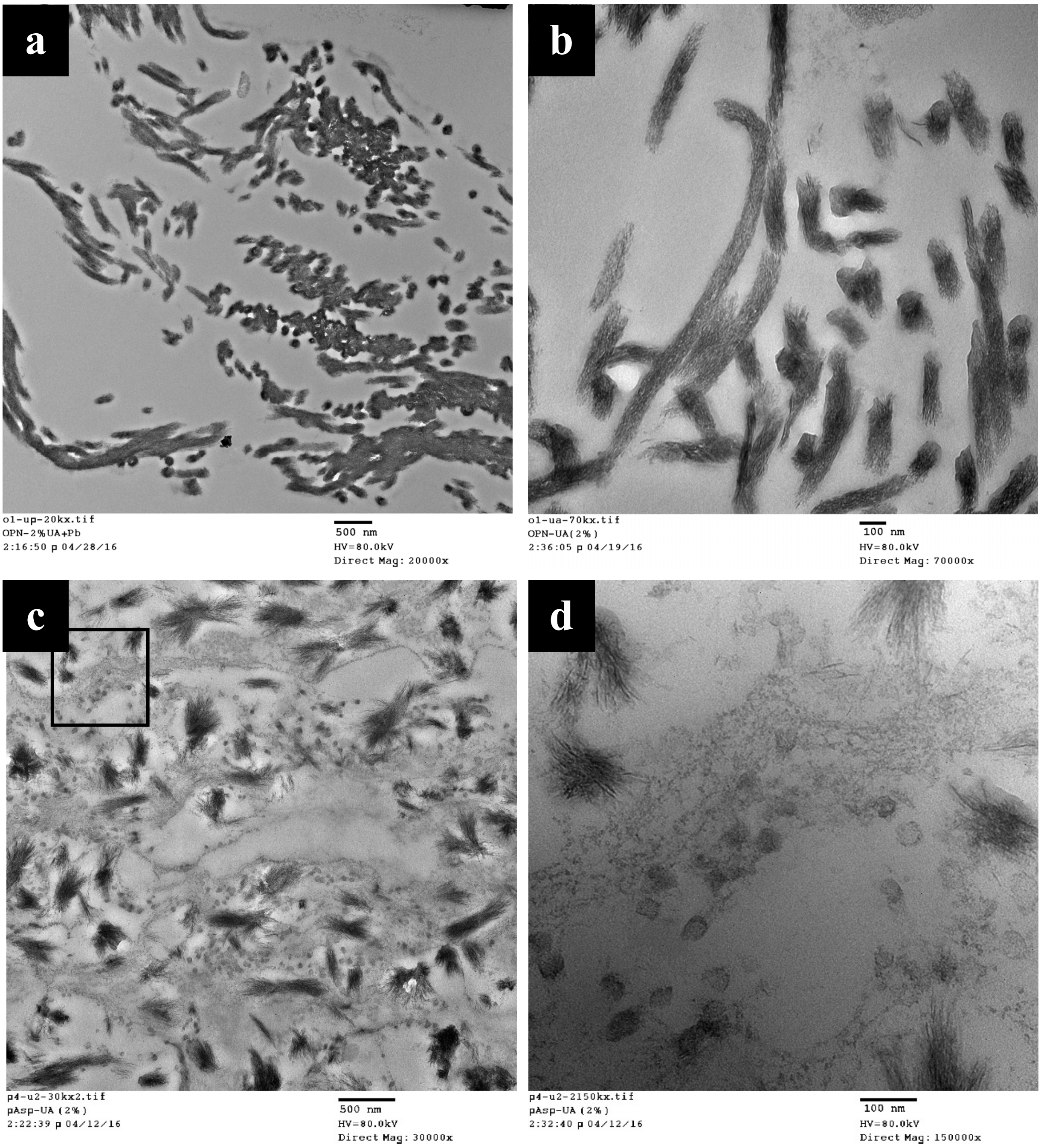

Figure S4. TEM micrographs of embedded, microtomed, and stained (2% UA) decellularized porcine kidney interstitial tissue that has been PILP-mineralized with OPN (top) and pAsp (bottom). These micrographs appear to show greater mineral confinement within fibrils in the OPN samples of (a) and (b), as compared to the splay of crystallites in the pAsp samples of (c) and (d). (d) When one zooms in on the box region in (c), an amorphous substance can be seen on and around the mineral crystallites (arrows), and given that it has a fluidic appearance, is likely a PILP-like phase that has accumulated on the collagen fibrils. The other circular entities below are cross-sections of fibrils, which appear to be in the early stages of amorphous mineral infiltration, and without the large agglomerates of phase and protruding crystallites. Reprinted with permission from ref. [46].

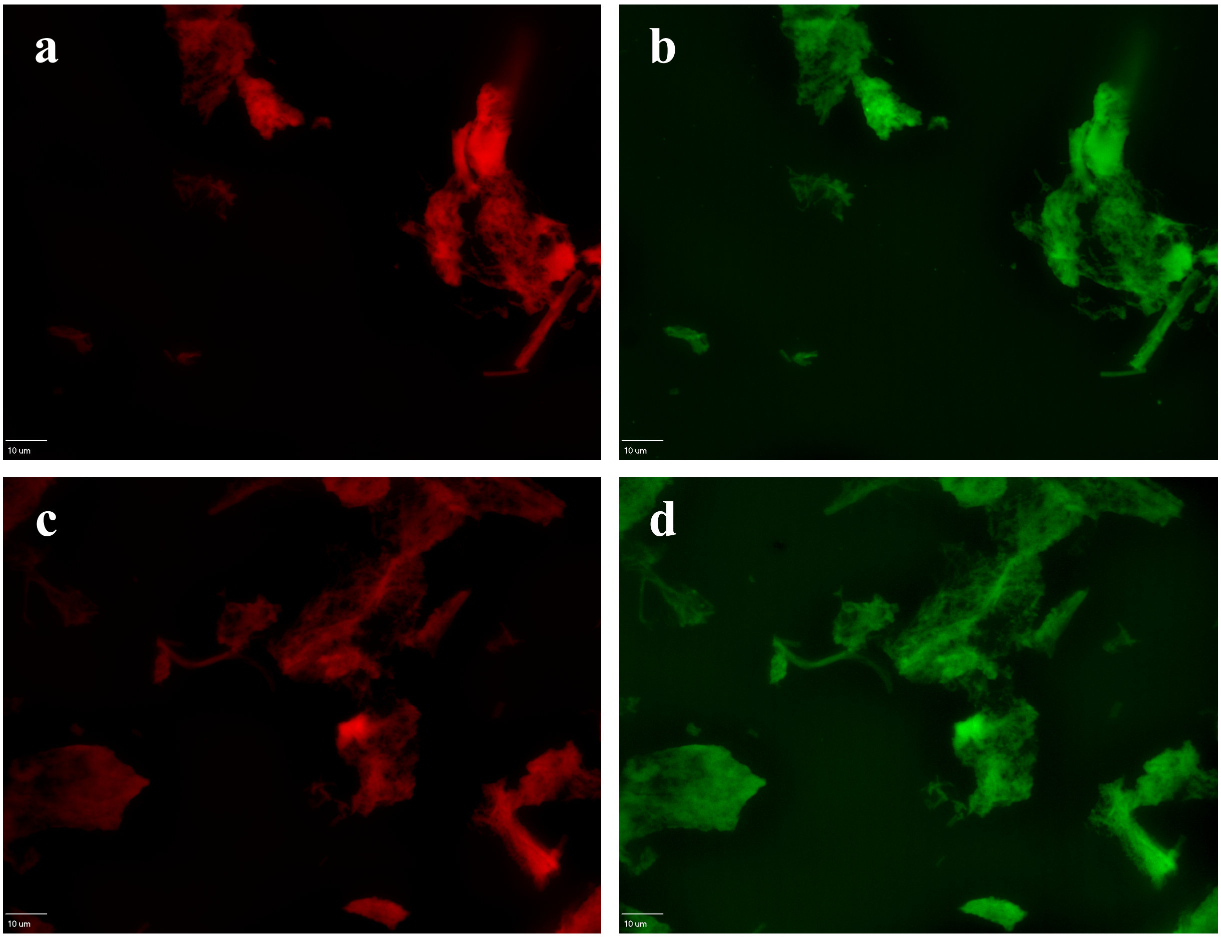

Figure S5. Projected views of confocal fluorescent z-stack images of collagen tagged with AlexaFluor® 647 (left) and process-directing agent tagged with FITC (right), after incubation in Tris buffer solution for 1 hour. The top row (a – b) shows OPN samples while the bottom row (c – d) shows pAsp samples. Scale bars are 10 μm.

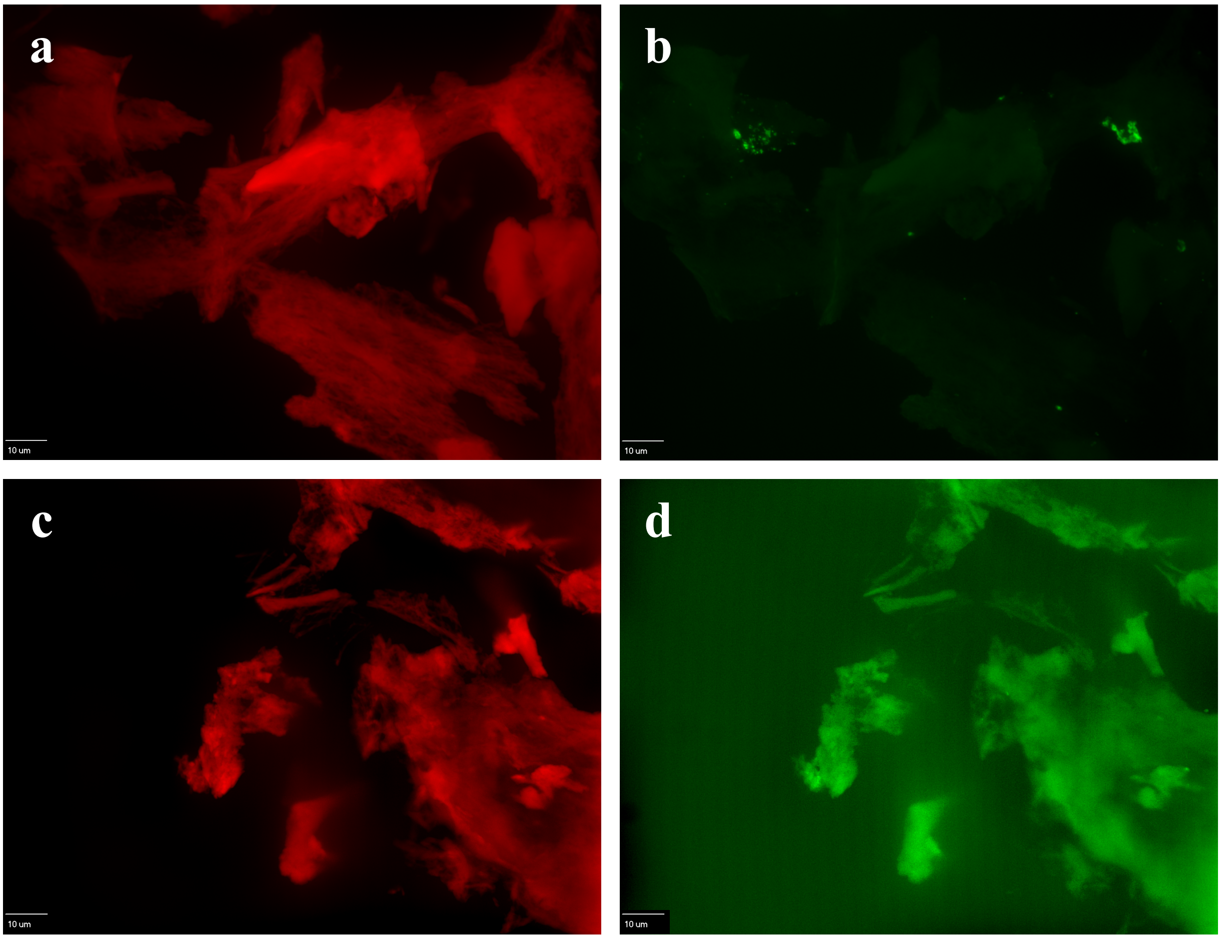

Figure S6. Projected views of confocal fluorescent z-stack images of collagen tagged with AlexaFluor® 647 (left) and process-directing agent tagged with FITC (right), after incubation in PILP solution for 1 hour. The top row (a – b) shows OPN samples while the bottom row (c – d) shows pAsp samples. Scale bars are 10 µm.

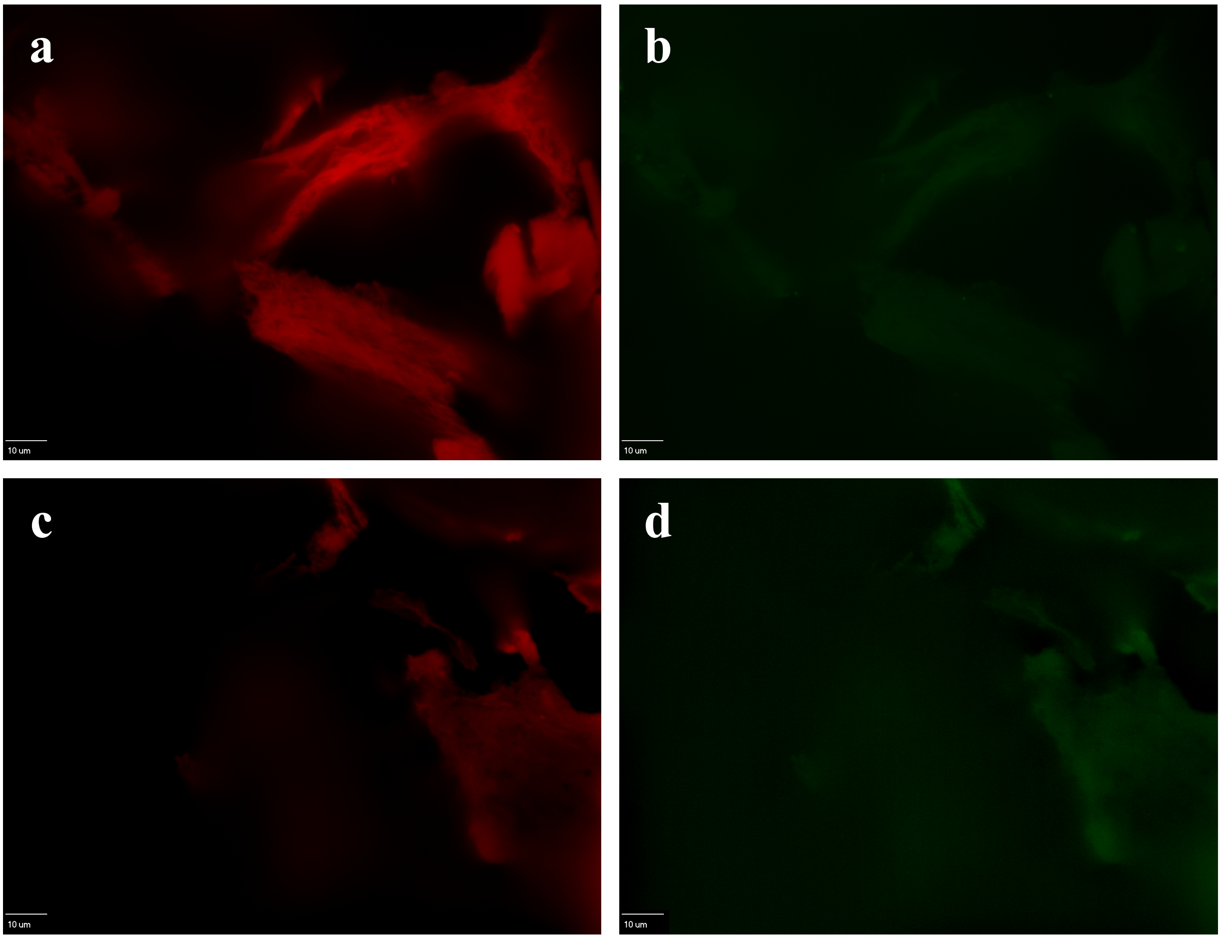

Figure S7. Individual confocal fluorescent images of collagen tagged with AlexaFluor® 647 (left) and process-directing agent tagged with FITC (right) after incubation in PILP solution for 1 hour. The top row (a – b) shows OPN samples while the bottom row (c – d) shows pAsp samples. Scale bars are 10 μm.

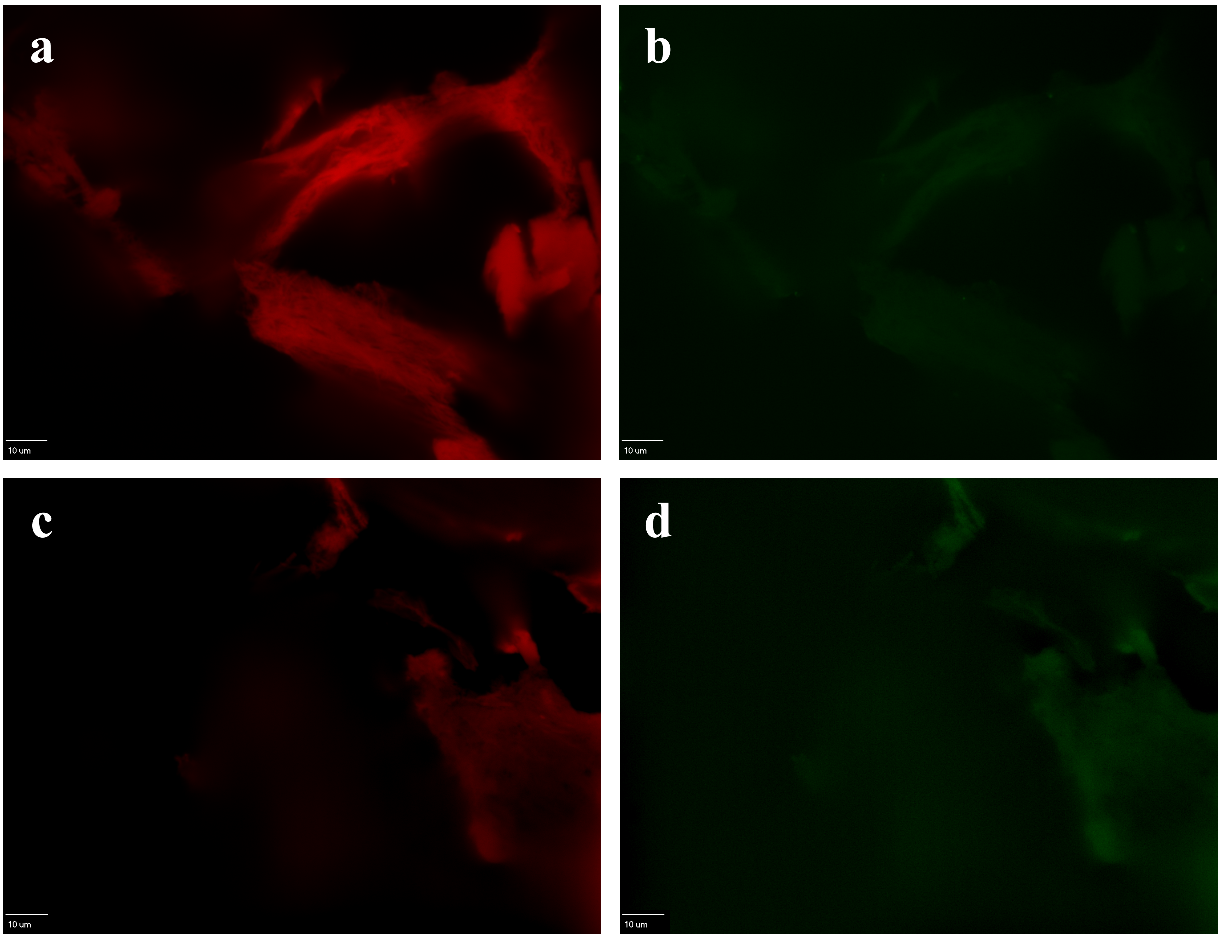

Figure S8. Individual confocal fluorescent images of collagen tagged with AlexaFluor® 647 (left) and process-directing agent tagged with FITC (right) after incubation in PILP solution for 1 hour. The top row (a – b) shows OPN samples while the bottom row (c – d) shows pAsp samples. Scale bars are 10 μm.

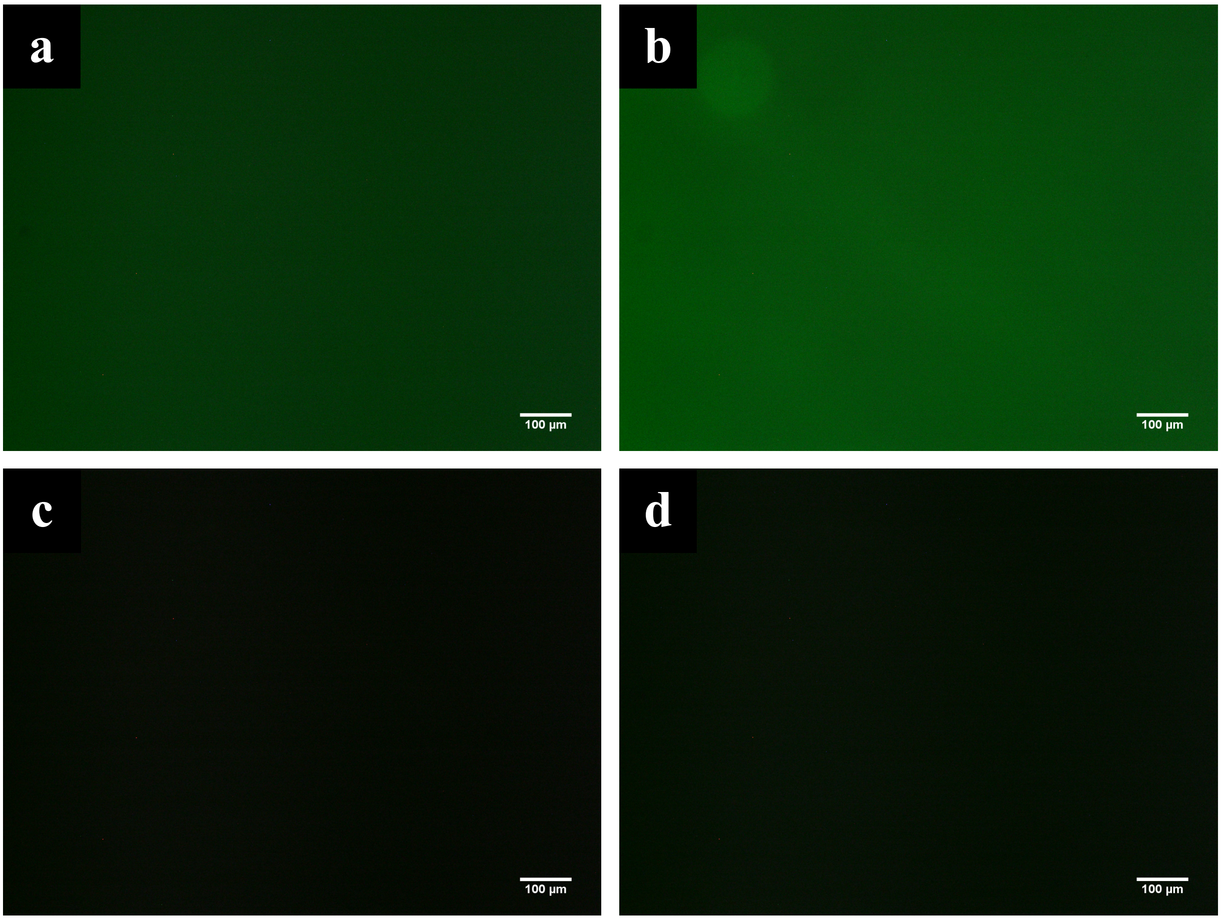

Figure S9. Epifluorescence images of glass slides incubated with FITC-tagged process-directing agent incubated in either Tris buffer (left) or PILP solution (right). The top row (a – b) shows OPN samples while the bottom row (c – d) shows pAsp samples. All images were taken with a 1500 ms exposure time and 15 gain. Scale bars are 100 µm.

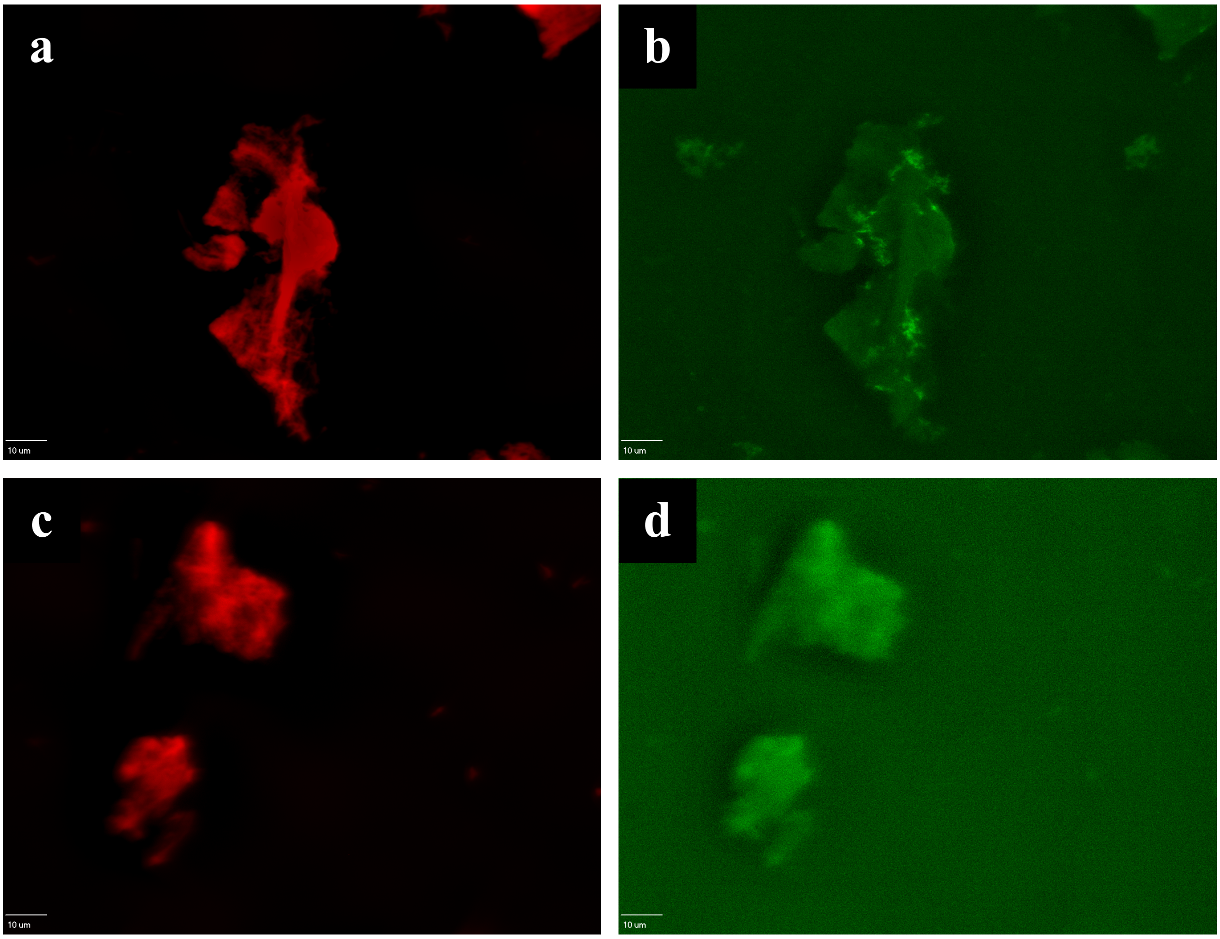

Figure S10. Projected views of confocal fluorescent z-stack images of collagen tagged with AlexaFluor® 647 (left) and process-directing agent tagged with FITC (right) after incubation in a larger volume (20 ml) of Tris buffer solution for 6 hours. The top row (a – b) shows OPN samples while the bottom row (c – d) shows pAsp samples. Scale bars are 10 µm.

Supporting Information: Videos

Video S1: OPN droplets visualized with NanoSight particle tracking analysis

Video S2: pAsp droplets visualized with NanoSight particle tracking analysis

Video S3: Droplets without polymer stabilizer visualized with NanoSight particle tracking analysis
